# An optimized location service for the fifth generation VANETs inspired by traffic lights

**DOI:** 10.1371/journal.pone.0259060

**Published:** 2021-11-18

**Authors:** Esraa Al-Ezaly, Ahmed Abo-Elfetoh, Sara Elhishi

**Affiliations:** Information Systems Department, Faculty of Computers and Information, Mansoura University, Mansoura, Egypt; University College of Engineering Tindivanam, INDIA

## Abstract

Vehicular ad-hoc networks (VANETs) address a steadily expanding demand, particularly for public emergency applications. Real-time localization of destination vehicles is important for determining the route to deliver messages. Existing location administration services in VANETs are classified as flooding-based, flat-based, and geographic-based location services. Existing localization techniques suffer from network disconnection and overloading because of 5G VANET topology changes. 5G VANETs have low delay and support time-sensitive applications. A traffic light-inspired location service (TLILS) is proposed to manage localization inspired by traffic lights. The proposed optimized localization service uses roadside units (RSUs) as location servers. RSUs with the maximum traffic weight metrics were chosen. Traffic weight metrics are based on speed of vehicles, connection time and density of neighboring vehicles. The proposed TLILS outperforms both Name-ID Hybrid Routing (NIHR) and Zoom-Out Geographic Location Service (ZGLS) for packet delivery ratio (PDR) and delay. TLILSs guarantee the highest PDR (0.96) and the shortest end-to-end delay (0.001 s) over NIHR and ZGLS.

## 1. Introduction

Owing to their continually increasing demand, particularly for public emergency applications, VANETs are an interesting area of investigation. Information is sent to the driver to prevent accidents, reduce traffic jams, and for entertainment purposes [1]. An intelligent warning message was employed in a study to reduce the number of vehicles, quick acceleration. Intelligent warning messages are sent via the VANETs. [2]. Reinforcement learning assists vehicles in determining the best route on their own [3].

Stable location services are important because of their continually increasing demand, particularly for public emergency applications. With tiny 5G cells and data centers, especially in dense urban scenarios, services can be quicker and more responsive. Vehicles can communicate with each other and roadside units using VANETs. In wireless ad-hoc networks (WANETs) and mobile ad-hoc networks (MANETs), nodes move without a paved roadway in all directions, whereas in VANETs, nodes are vehicles that are constrained to the paved roadway in VANETs [4]. Because the topology varies in VANETs, the position, speed of vehicles, and road type should be considered to provide location information [5].

Scalable location services are important for covering all types of roads, including primary roads and subways. Big traffic data processing as a VANET challenge was handled using fog computing [6]. Unlike earlier ad-hoc networks such as WANET and MANET, VANETs provide infrastructure-based communication [7]. VANETs have many types of communication: vehicle-to-vehicle (V2V), vehicle-to-infrastructure (V2I), and infrastructure-to-infrastructure (I2I). V2V communication enhances driving by disseminating using the dissemination of information to vehicles in their vicinity. Road-side units are utilized in our proposed location service to cover four directions at the intersections. All types of communication in VANETs were exploited.

In this study, traffic-light-inspired location service was used to help define the location of vehicles inspired by traffic lights. Stability was supported using a stable roadside unit neighboring the traffic lightbox. Scalability was supported using roadside units covering four directions at the intersections. Traffic lights serve both types of roads, the main road which that has four directions, and the subway which contains four directions. Each direction had a traffic lightbox. The red wait time for the green light status was smaller in the primary roads. This means that green status time in the primary road is more than that in the secondary road.

A few location servers are used in our location administration service to minimize the delay and overhead. Our well-distributed location service in the four road directions enables location information distribution, precise results of vehicle locations, and small overheads. Location administration services located at intersections are robust because of vehicles’ low speeds and stops. We tested our traffic-light-inspired location administration service by varying the number of vehicles and their speeds. The results show that this is an economic place and scenario. The reminder of this paper is structured as follows: Section 2 presents the related work, and Section 3 shows the proposed TLILS. The results of the TLILS are verified and presented in section 4. Section 5 concludes with future work and conclusions.

## 2. Related works

Vehicle dissemination in VANETs has two types: group-based and position-based. Messages are distributed to a collection of vehicles at various locations using group-based dissemination protocols [8]. An article introduced a new classification system for information dissemination protocols based on the application types. This work presented the first integrated dissemination for location- and group-based VANET applications [9].

The security of vehicular network messages is an urgent issue because it allows the use of s-called m-government and internet of things [10]. Despite security threats, uniform coding standards, avoiding conflict collision, security, privacy protection, and trust management were implemented to address these concerns [11]. For VANET, an authentication method in which the sender and receiver of the message are validated was proposed. The sender digitally signed the message with a public/private key, and the message was decrypted by the recipient. The sender and receiver of the message were validated [12]. Batch verification was used to support the security of traffic data. The RSU simultaneously verified the validity of the signatures from multiple vehicles [13]. To reduce latency, unmanned aerial vehicles assist in the identification and routing of harmful vehicles [14].

Using vehicular cloud computing and body area networks, driver sickness and sleepiness behind the wheel were detected [15]. Authentication of emergency vehicle protocols was efficient against attacks such as impersonation, device theft, and reputation attacks [16]. Batch authentication and key exchange schemes were proposed for the 6G VANET to provide high-level security by avoiding communication with malicious vehicle users. To ensure privacy, message alteration is prohibited during transmission [17]. A cache decision algorithm enables the decisions to use a secure cloud connection or for fast and low secure fog connections [18]. These studies lack the optimal placement of the location servers.

Vehicles in the destination location only aid in transmission to the target area in position-based dissemination protocols. Positioning services are an urgent step for both types of dissemination protocols. Flooding-based, flat-based, and geographic-based services have been classified as existing location administration services in VANETs.

In flooding-based location services, several location updates messages concerning the number of hops is large [19]. In addition, NIHR relayed on limited flooding and did not rely on a location service required by other protocols proposed for VANETs [20]. It is unsuitable for vehicle-to-vehicle communication in dense cities. Node density increases because of flooding. It is suitable for frequent disconnecting networks.

Existing location services rely on vehicles for localization, reducing stability and causing disconnections. In the cache-based protocol, receivers of cache locations of nodes passing intersections act as location servers known as guideposts [21]. Flooding in this protocol increased the query and update overhead, particularly in areas with a high density of vehicles at intersections.

Zoom Out Geographic Location Service (ZGLS) was a protocol proposed for implementing a flat quorum-based location administration service for VANETs. To track destinations, a group of vehicles known as location servers was used [22]. In ZGLS, when the vehicle is on its way to location-based services, a nearby roadside unit informs the source about them. As a result, localization is intended for service providers rather than for vehicles. It did not address whether there is no nearby facility supplier in the road segments in roadside units.

Recent studies have focused only on V2V communication [23]. For location services, vehicles were used as moving location servers. Moving vehicles cause network disconnections. Stable location service management is required. A collaborative vehicle location management service for enhanced hybrid reactive and proactive multicast in VANETs was presented [24]. RSUs are exploited in cashing and location dissemination, as well as multicast message transmission. This service overcomes the problem of multiple location server changes, provides load distribution.

Previous Location service VANETs have problems. Vehicles are location servers that are not stable. The stable roadside unit rule is used for communication and sending messages text only, not for localization. Roadside units are numerous and distributed along with different locations which decrease efficiency and increases costs. Base stations are many and distributed which increases costs. Many network disconnections occur because of high-speed vehicles. To control traffic lights, internet of things-enabled light intensity was proposed to reduce light consumption by intelligently switching ON/OFF lightning and to inform faulty lamps. No communication between the vehicle and the traffic light was supported [25]. The proposed geographic-based location service for 5G VANETs inspired by traffic lights supports a stable and static localization near each traffic light. 5G enables faster management. Forwarding to each far vehicle from the traffic lights in the branches and subways. Therefore, the scalability is supported.

## 3. Traffic light-inspired location service

In this section, we present the localization problem, 5- traffic-based location service, and TLILS search.

### 3.1. Optimized 5G traffic-based TLILS choice

The optimized location server choice has many optimization parameters such as speed, time, and density:

#### 3.1.1. Speed

The optimized location services are stable. RSUs utilized as location servers are stable and the source vehicle is preferred to be stopping or at low speed for increasing connectivity.

#### 3.1.2. Time

The source connection time is preferred to be suitable for search message transmission, acknowledgment, and reply. The optimized location server is the RSU with the highest source’s connection time.

#### 3.1.3. Density

Many source vehicles and forward vehicles are preferred to increase connectivity. For location service optimization, the RSU with the largest number of neighbors is chosen.

Given a road network *N (I*, *B)* and a set of RSU sites *L (1*, *2…*, *N)*, solve the following problem. The number of cars passing through intersection *I* at a given time is denoted by *N*. The average speed of cars going through intersection *I* is denoted by the letter *S*. The source connection time *T* is the average of the periods during which the source continues to send beacons to RSU *I*. The traffic weight metric *W*_*I*_, which indicates how well intersection *I* functions as TLILS, is denoted as follows:

$$W_{I}=\frac{N}{S\times T}$$ (3.1.1)

RSU and the neighbor are compared, and the optimized weight is the chosen as the maximum traffic weight metrics:

$$TLILS=\text{max}\{W_{I},\;W_{I++}\}$$ (3.1.2)

**Fig 1:**
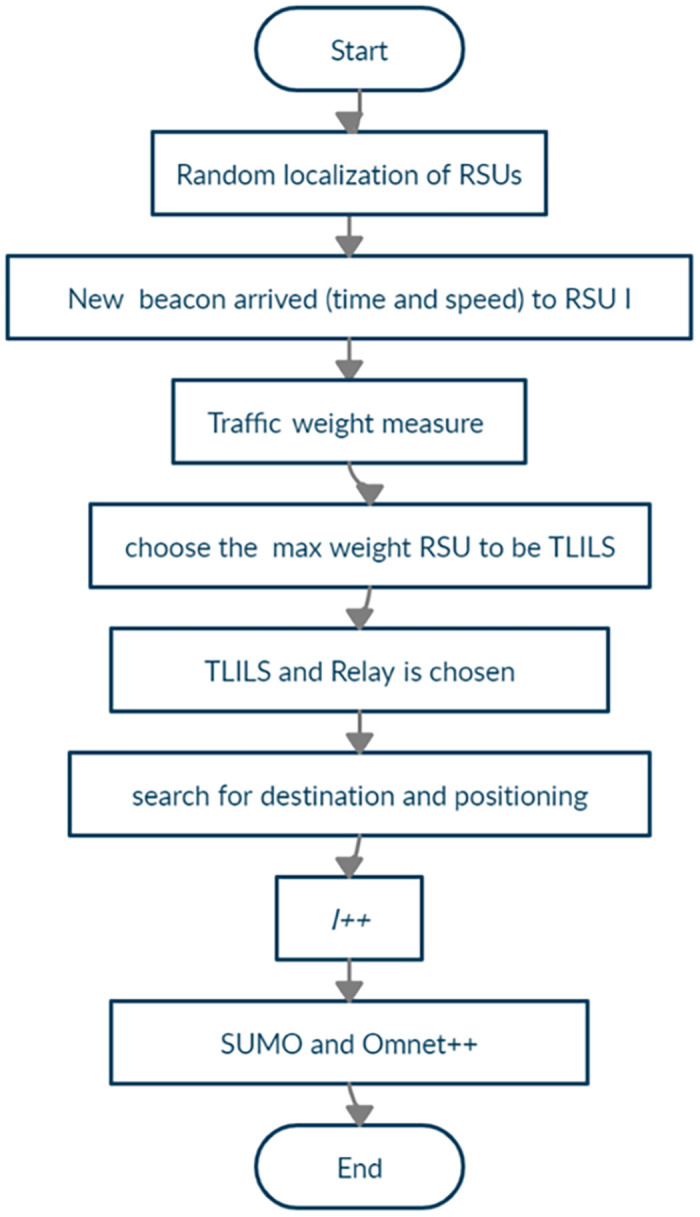
The execution procedure for the optimized 5G traffic-based location service.

The flowchart in Fig 1 shows the traffic-based location-server placement algorithm. Once on beacon arrived from neighbour vehicle to RSU, the beacon information is stored in RSU table. The traffic weight in Eq 3.1.1 is computed for each RSU at the intersection. The RSU weight is compared to the neighbor RSU and the maximum RSU is chosen ad TLILS.

**Fig 2:**
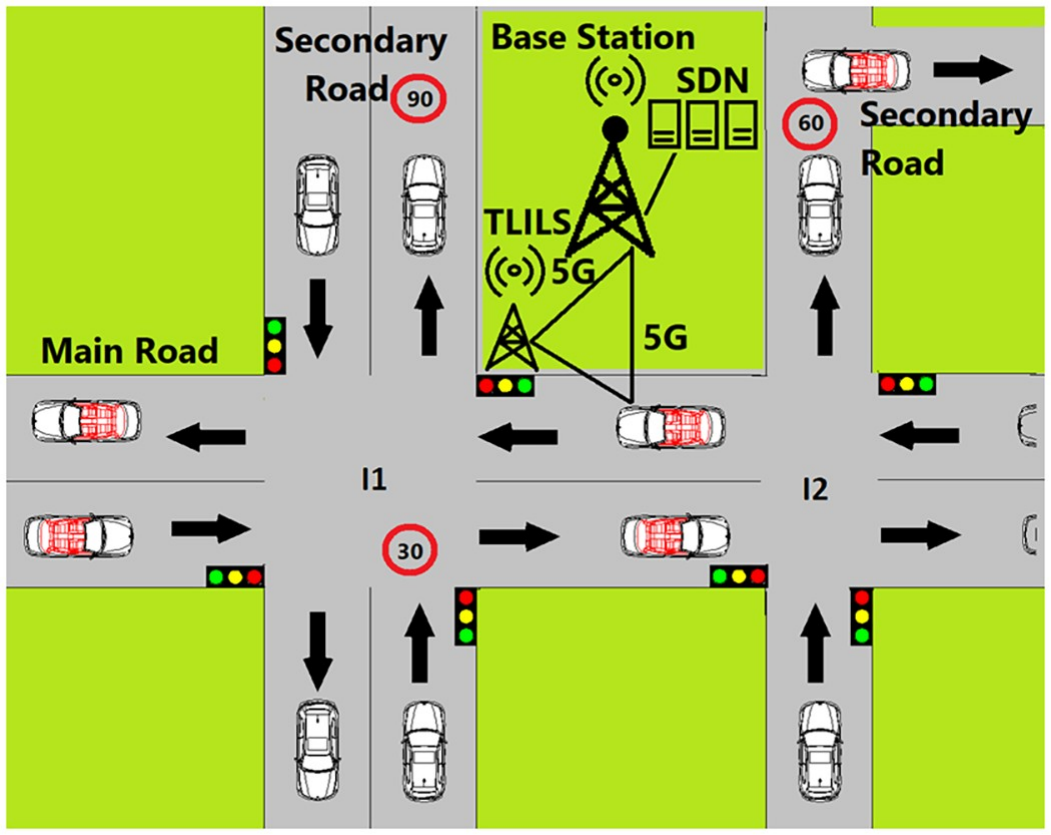
Optimized 5G traffic-based location server placement.

**Fig 3:**
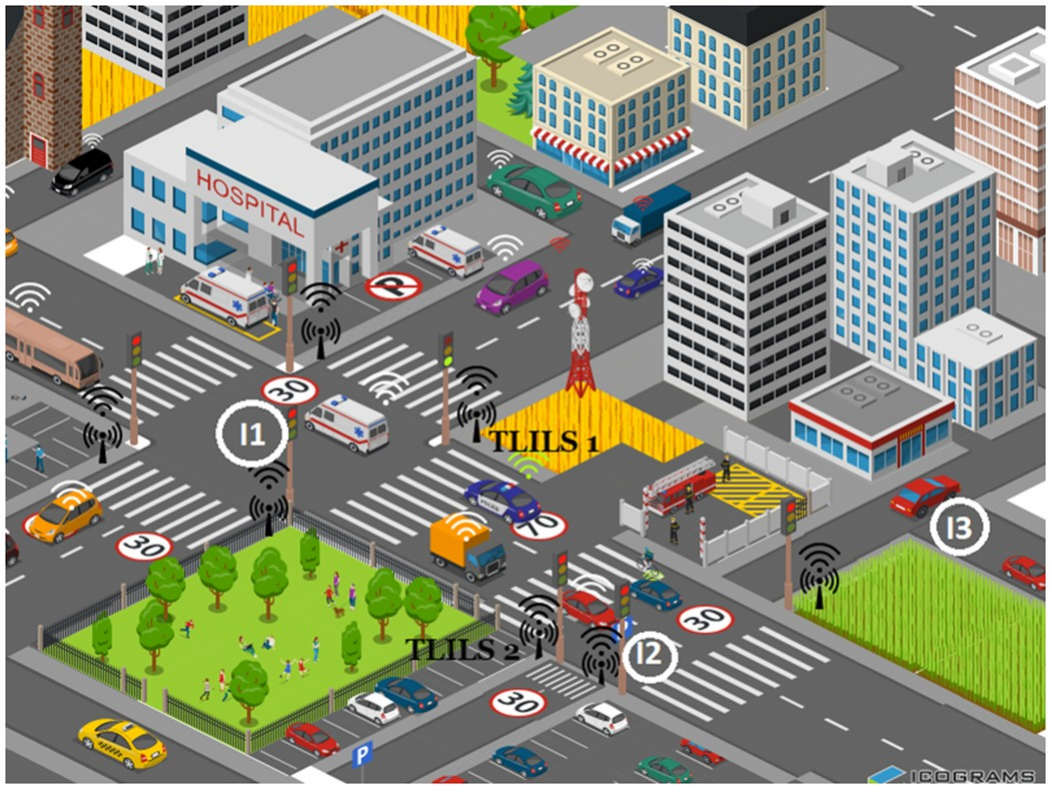
3D design of location service for VANETs inspired by traffic lights.

TLILS is stable as the vehicles are slow at intersections as shown in Figs 2 and 3. It is also scalable because all cars passing through the four directions are localized. Vehicle and roadside units exploit forwarding to each far vehicle on both primary roads and subways. Information dissemination is supported using roadside units that localize vehicles in four directions.

In Fig 2, 5G is combined with VANET. Software-defined networking (SDN) is enabled for base stations. The SDN-based VANET system helps to decrease transmission delay. In Fg.3, Tl is placed at the intersection where there is a road or more in two directions. If one road only at the intersection has two directions, three TLs are built as in *I*2. If the two roads at the intersection have two directions, four TLs are built as in *I*1. One-way roads have no traffic lights as in *I*3. There is no need for a traffic light in *I*3 because the secondary road is on way. Pedestrians passing through the main road will increase accidents when the traffic light at intersection *I*2 is green as the vehicles accelerate. Pedestrians passing through the secondary roads will increase accidents because vehicles must be informed early before stops. However, there is a pedestrian pass 100 m after the secondary road.

As TLILS prefers to be located at TL stops, TLILS mimics traffic lights. One RSU at the intersections is utilized such as TLILS1 and TLILS 2 and other RSUs are the relays. The middle relay is utilized to broaden the coverage region of interest. When TLILS 1 and 2 are compared using traffic weights, TLILS 2 is chosen. The overhead should be reduced by using the load distribution at the intersection. The overhead decreases at a high number of vehicles as a search query for location and messages are sent in the same transmission. Using 8, or 16 TLILSs which increases the overhead particularly at high speeds of vehicles because of the large errors in routes. TLILS uses roadside units to help forward message dissemination and positions directly to the destination vehicle which reduces the overhead.

Table 1 displays the selection of rows from the location information table cached in the primary TLILS (PTLILS). The table contains the information recording time (seconds) for the recorded vehicle number, as well as the location of the source vehicle of the beacon message at x, y, and z coordinates, speed, road number, and road type. The notations used in Algorithm 1 are presented in Table 3.

**Table 1:** Example row from TLILS passing location table [1].

Time (seconds)	Vehicle number	Location (x, y, z)	speed (m/s)	Road type
15	N1	1500,1500,0	10	primary
16	N2	1500,1600,0	15	primary
17	N1	1500,1500,0	0	primary

Table 1 shows an example row from a TLILS passing location table.

Table 2 shows the selection of rows from the location information table cached in the secondary TLILS. TLILS [0] is chosen because of Algorithm 1.

**Table 2:** Example row from TLILS passing location table [2].

Time (seconds)	Vehicle number	Location (x, y, z)	speed (m/s)	Road type
15	N3	1500,1500,0	40	secondary
16	N4	1600,1600,0	50	secondary

The notations used in Algorithm 1 are presented in Table 3.

**Table 3:** Notations used in Algorithm 1 and their description.

Notation	Description
*ACK*	Acknowledgment
*CD*	Connection duration
*TLILS*	Traffic Light-Inspired Location Service
*L*	Number of RSUs
*N*	Number of vehicles recorded
*S*	Speed
*Sum*	Summation of speeds
*CDs*	Summation of connection duration

In Algorithm 1, the decision-making technique is discussed for choosing the location server. In step 2, a new beacon is sent from the neighbor vehicle and the RSU replies in step 3 with an acknowledgment. The beacon contains the beacon time and speed of the vehicle, location, and road type of the vehicle. In step 4, the connection time is computed. From steps 5 to 20, for each RSU and each neighboring vehicle, if the vehicles stayed connected for some seconds, or if the neighbor vehicle stopped, the average speed is calculated in steps 8 and 15. In step 17, the weight metric *W*_*I*_, which indicates how well intersection *I* functions as TLILS, is computed. In step 18, the maximum weight is chosen. Otherwise, another RSU was tested for the optimization.

**Algorithm 1**: Optimized 5-G Traffic-Based Location Server Placement

1: **Start**

2: A New beacon arrives (recording time, speed, location, road type) to RSU

3: RSU replies with *ACK*

4:   CD = Recording end time—Recording start time

5: **For l** = 1,2, …*L*

6:  **For** *n* = 0,1, …*N*

7:   **If** *CD*> = 2 OR *s*_*n*_ = 0

8:    *Sum***+ =** *s*_*n*_

9:    **CDs+ = CD**

10:    **Return** *sum*

11:   **End if**

12:  *n*++

13:  *return n*

14: **End for**

15:   **If** *n***>** = 3

16:    average *s = sum/n*

17:    **If** average *s*< = 30

18     **T = CDs/n**

19:    *W*_*I*_ *= N/* average s *T

20:     *TLILS = max {RSU[l]*, *RSU[l++]}*

21:    **Else**

22:    *l*++

23:    **End if**

24:   **End if**

25: **End for**

26: **End**

### 3.2. TLILS search

After the TLILS choice, the search query message is sent using the 5G-VANET connection. The functioning steps of the positioning vehicles in TLILSS are presented in Algorithm 2. From steps 2 to 23, the positioning of recently connected TLILSs uses location administration to find vehicles in TLILSs. The notations used in Algorithm 2 are listed in Table 4.

**Table 4:** Notations used in Algorithm 2 and its description.

Notation	Description
*SR*	Source vehicle region
*D*	Number of destinations in the group member table
*TLILS*	Traffic Light-Inspired Location Service
*A*	An acknowledgment Message
*S*	The message

**Algorithm 2** Destination Position Search in TLILSS:

1: **Start**

2: **For d** = 0,1, …*D*

3:  **If** (*d* is in *SR*) **then**

4:   Transmit message straightly to *d* and *d* respond with an *A*

5:  **Else if** (*d* is NOT in *SR* OR No *A*) **then**

6:   **For** l = 0,1, …4 **do**

7:    Send a search question to TLILS [l] about d and S.

8:    **If** (*n* is found now) **then**

9:     Transmit *S* using TLILS[l] and relay.

10:     *d*++

11:    **Else if** (*d* is found before) **then**

12:     l++

13:     *d*++

14:    **Else**

15:     l++

16:     End for

17:    **End if**

18:   **End if**

19:   *d*++

20: **End for**

21: Repeat using delivery a seek query about d and S using neighbor vehicles to TLILSs in the next intersection.

22: Remove S

23: **End**

When a destination passes beside the source vehicle, the source examines the id for destinations. If a beacon is sent from the destination vehicle to the source vehicle, it means the destination is within the source’s wireless communication range, and in step 4, the message is directly transmitted from the source vehicle to the target vehicle. When the messages are received by the destination vehicle, it sends an acknowledgment message to the source, and the destination id is updated. In step 7, if the source vehicle does not receive an acknowledgment message after a reasonable amount of time, a seek inquiry is sent to the final TLIS.

As the destination vehicle is currently recorded in step 9, the message is sent directly to the current recipient of the destination vehicle. An acknowledgment is sent from the destination vehicle in step 9. The working step of the positioning of the non-recorded or earlier-recorded destination vehicle using the next TLILSS at the intersection is shown in step 12. The query is sent to the neighbor TLILS. If the destination vehicle is not found, in step 21, the algorithm is repeated using delivery a seek query about d and the message using neighboring vehicles to TLILSs in the next intersection.

## 4. Simulation model

In this section, the proposed simulation parameters, metrics, and the two scenarios are proposed. The network design was drawn using icograms. The presented simulation uses an objective modular network tested in C++ (OMNET++) for 5G VANET simulations [23]. OMNET++ uses the simulation of urban mobility (SUMO) which generates vehicles and road maps. The simulation parameters are described in the next section.

### 4.1. Simulation parameters

In Table 5, the TLILS tested on SUMO has 15 intersections. TLILS was tested using eight TLISs placed at 15 intersections. The TLILS was also tested using four TLISs. In addition, 16, and 32 TLISs were more than the number of intersections tested.

**Table 5:** Parameters for simulation to find the traffic light-inspired location service for VANETs.

Simulation parameter	Value
vehicles on the road	20, 50, 100, 150, and 200
Vehicles’ speed in meters per second (meters/second)	10
Range of wireless transmission (meters)	200
Dimensions of the playground (meters)	2499 ×2499
Time limits for simulations (seconds)	5590
Intervals between beacons (seconds)	1
Total number of TLILSs	4, 8, 16, and 32
The number of crossroads	15

### 4.2. Simulation’s metrics

Simulation metrics are such as packet delivery, packet loss, delay, and overhead.

#### 4.2.1. Packet delivery ratio

PDR is the ratio of the succeeding packets received by the destination vehicle to the total number of packets sent by the source vehicle. PDR is calculated using Eq 4.2.1 [26]:

$$PDR=\frac{R}{S}$$ (4.2.1)

*R* is the received packets is the number of packets successfully received by the destination vehicle, and *S* is the total number of packets sent by the source vehicle.

#### 4.2.2. Packet loss ratio

The packet loss ratio is the number of lost packets divided by the total number of packets sent [27]. All packets have the most time before they must be executed, and if they do not execute, packet loss is minimized [28, 29]. The PLR is calculated using the following equation [26]:

$$PLR=\frac{\left(\text{S}-\text{R}\right)}{\text{S}}$$ (4.2.2)

*S* denotes the number of packets sent by the source vehicle, and R denotes the number of packets received by the destination vehicle.

#### 4.2.3. End-to-end delay

The time it takes for a packet to travel from a source vehicle to a destination vehicle via a vehicular network is referred to as the delivery delay. This delay time is caused by routing process delays, including routing discovery, routing transmission, re-transmission, interface delay, and MAC delay [30]. PLR lengthens the end-to-end delay owing to the re-transmission of failed packets, leading to an increase in the end-to-end delay [30]. The end-to-end delay is calculated by subtracting the source transmission time from the arrival time to the destination. In Equation 4.4, the average delay for all packets was calculated [26]:

$$\text{Average}\;end-to-end\;delay=(\sum_{p=1}^{n}\left(\;T_{R}-T_{S}\right))/\text{n}$$ (4.2.3)

#### 4.2.4. Overhead

Text messages are combined with many types of overhead. The authentication overhead caused by the authentication method in 5G VANETs is identified in the analysis [31]. Normal routing overhead, authentication overhead, and control overhead were also added to the text messages. The following equation is used to compute the overhead of all nodes:

$$\text{Overhead}=\sum_{v=1}^{m}C_{v}$$ (4.2.4)

*C*_*v*_ denotes the number of additional bytes transmitted by vehicle (v), and m denotes the number of cars.

### 4.3. Simulation scenarios

The average rates were calculated for each case. In Scenario 1, the effect of increasing vehicle speeds on the dissemination performance utilizing TLILS, NIHR, and ZGLS was investigated. With an average speed of 10 m/s, we tested the impact of a varied number of cars on TLILS performance. The impact of adjusting the amount of TLILSs in Scenario 2 utilizing 4, 8, 16, and 32 TLILSs was tested at 15 intersections.

## 5. Experimental results

A comparison between TLILSs using eight TLISs placed at 15 intersections. TLILS is placed at the intersection which is inspired by traffic lights. Four TLISs, which are lower than the number of intersections, were used in the test. There are 16 to 32 TLISs tested, which is more than the number of intersections. A comparison of the results of ZGLS, NIHR, and TLILS is shown in Scenario 1 to test stability.

### 5.1. Comparison between TLILS, BBR, and ZGLS

Scenario 1 depicts the influence of a variable number of cars on TLILS, ZGLS, and NIHR performance. There were 20, 40, 80, 200, 400, and 800 vehicles in the simulation, at an average speed of 10 m/s and 8TLILSs.

**Fig 4:**
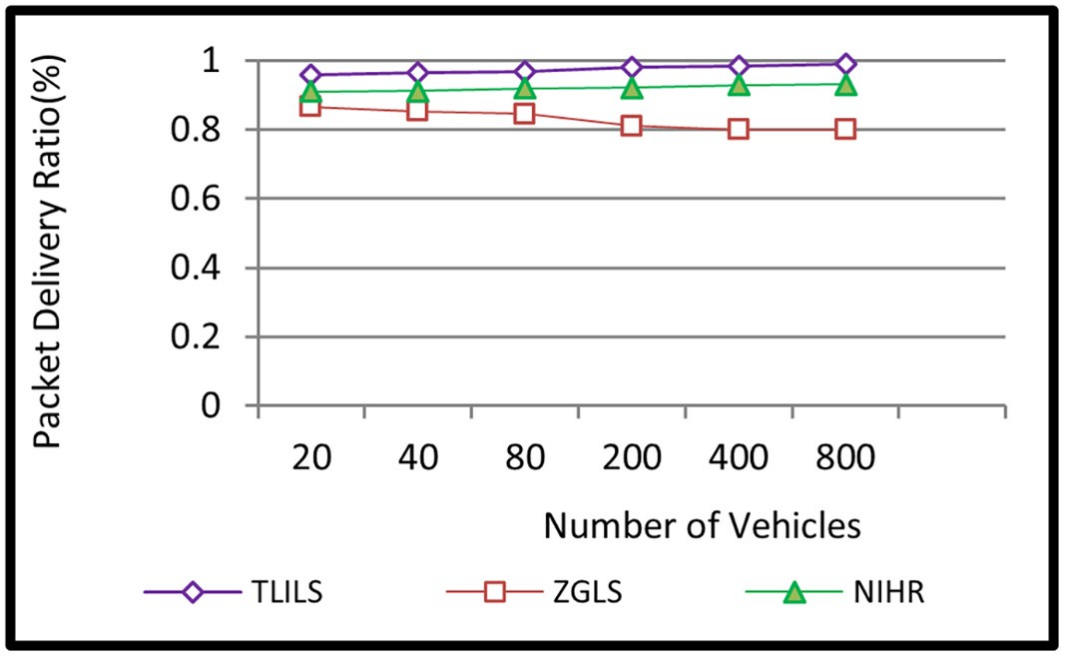
Information dissemination packet delivery ratio using the TLILS, ZGLS, and NIHR.

Because TLISs provide stability, they have the highest PDR (98 percent). This is because the location servers are stable. TLILS beats NIHR because NIHR broadcasts the message to every neighbor to forward it to the destination which increases packet overhead. PDR increases as the maximum density increases from 20 to 800 cars as shown in Fig 4. The PDR in NIHR is less than 0.90 because the location service used to determine the position of the destinations is unstable. Because the PDR in ZGLS is less than 0.8, it relies on moving cars as location servers, which reduces stability.

**Fig 5:**
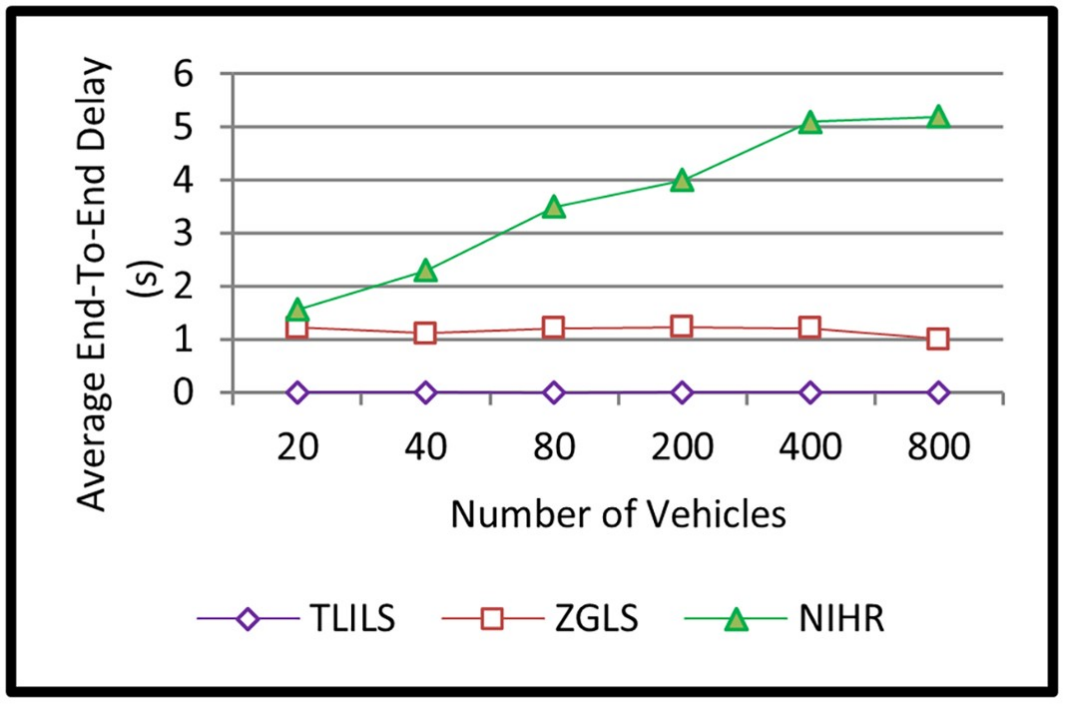
Information dissemination delay of the TLILS, ZGLS, and NIHR.

TLILS has the shortest delay as shown in Fig 5 (smaller than 2 ms). This is because TLISs find positions using forwarding messages to destinations and exploiting optimized weighted RSUs as location servers. In NIHR, the end-to-end delay is more than two seconds, especially in a high number of vehicles because vehicles use flooding in all directions. Because of the storage time and space of earlier information, the end-to-end delay in ZGLS is lower than two seconds in many vehicles, as shown in Fig 5. This is quite low because ZGLS employs a flat quorum-based location administration service, and a nearby roadside unit notifies the source of its existence.

**Fig 6:**
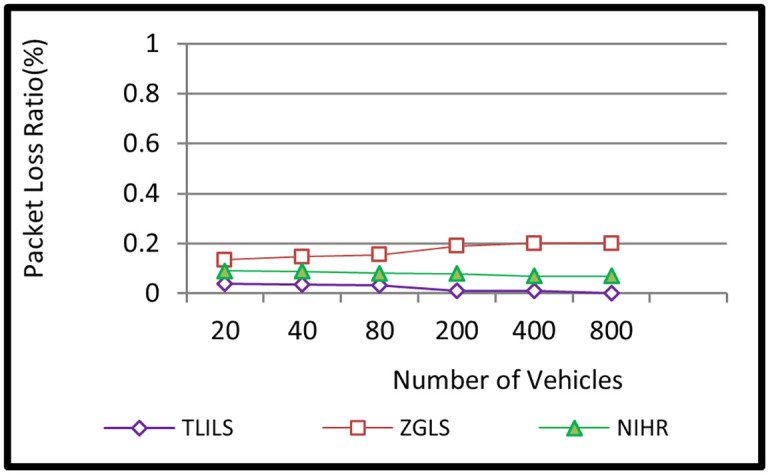
Information dissemination packet loss ratio using the TLILS, ZGLS, and NIHR.

Because TLISs provide stability, they have the lowest PLR (0.02 percent). This is because the location servers are stable. TLILS beats NIHR because NIHR broadcasts the message to every neighbor to forward it to the destination which increases packet overhead. PLR decreases as the minimum density decreases from 20 to 800 cars as shown in Fig 6. The PDR in NIHR is larger than 0.1 because the location service used to determine the position of the destinations is unstable.

**Fig 7:**
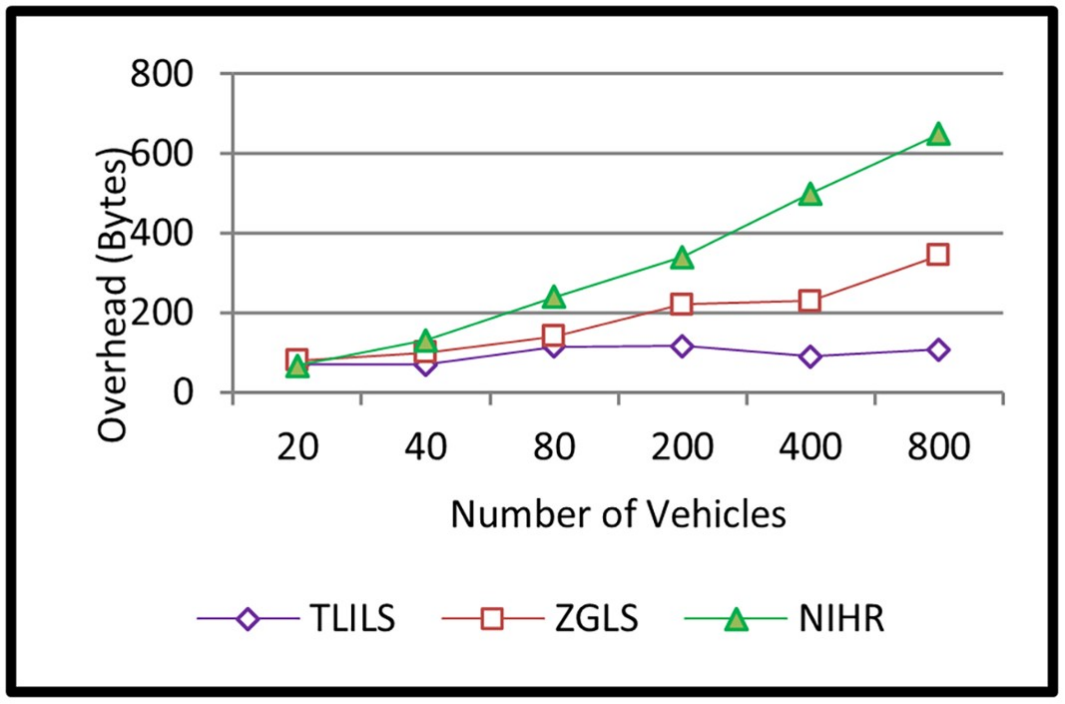
Information dissemination overhead of the TLILS, ZGLS, and NIHR.

TLILS has the smallest overhead as shown in Fig 7 (smaller than 100 bytes). This is because TLISs is exploiting optimized weighted RSUs as location servers. In NIHR, the overhead is more than 100 bytes, especially in a high number of vehicles because vehicles use flooding in all directions. Because of the storage time and space of earlier information, the overhead in ZGLS is more than 100 bytes in many vehicles, as shown in Fig 7. This is quite low because ZGLS employs a flat quorum-based location administration service, and a nearby roadside unit notifies the source of its existence.

**Fig 8:**
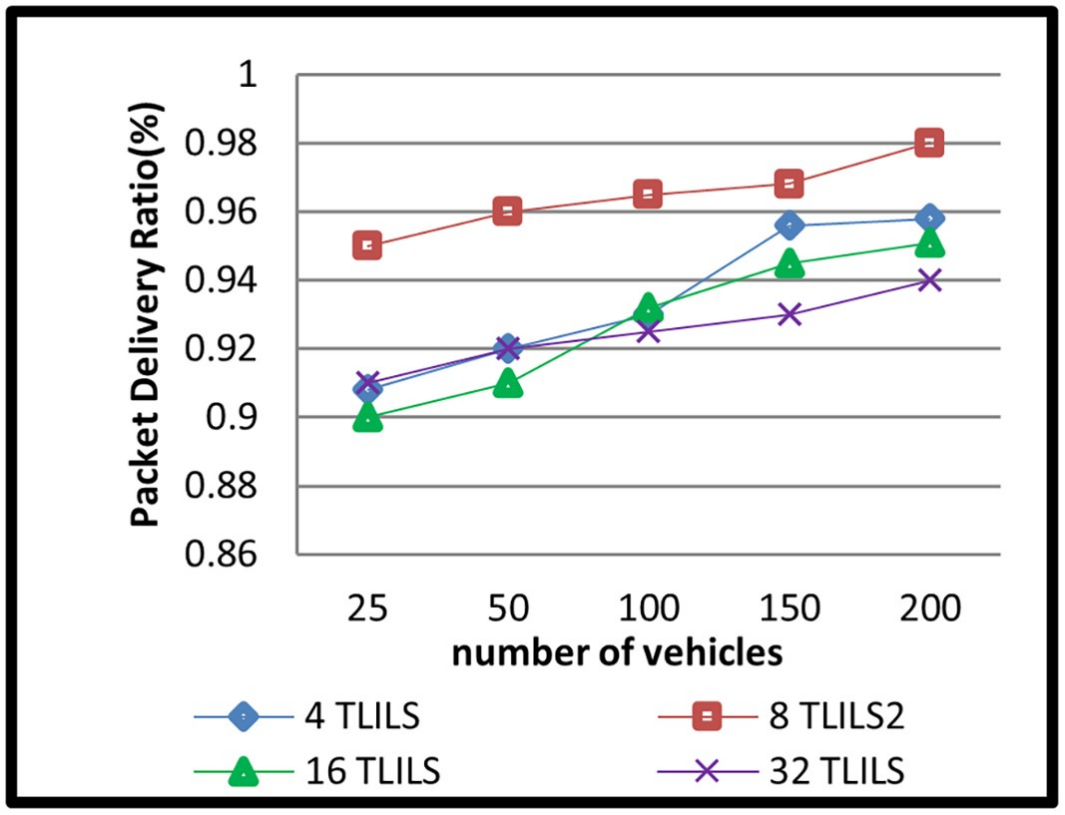
Packets delivery ratio of information dissemination with the TLILS with 10 m/s speed.

**Fig 9:**
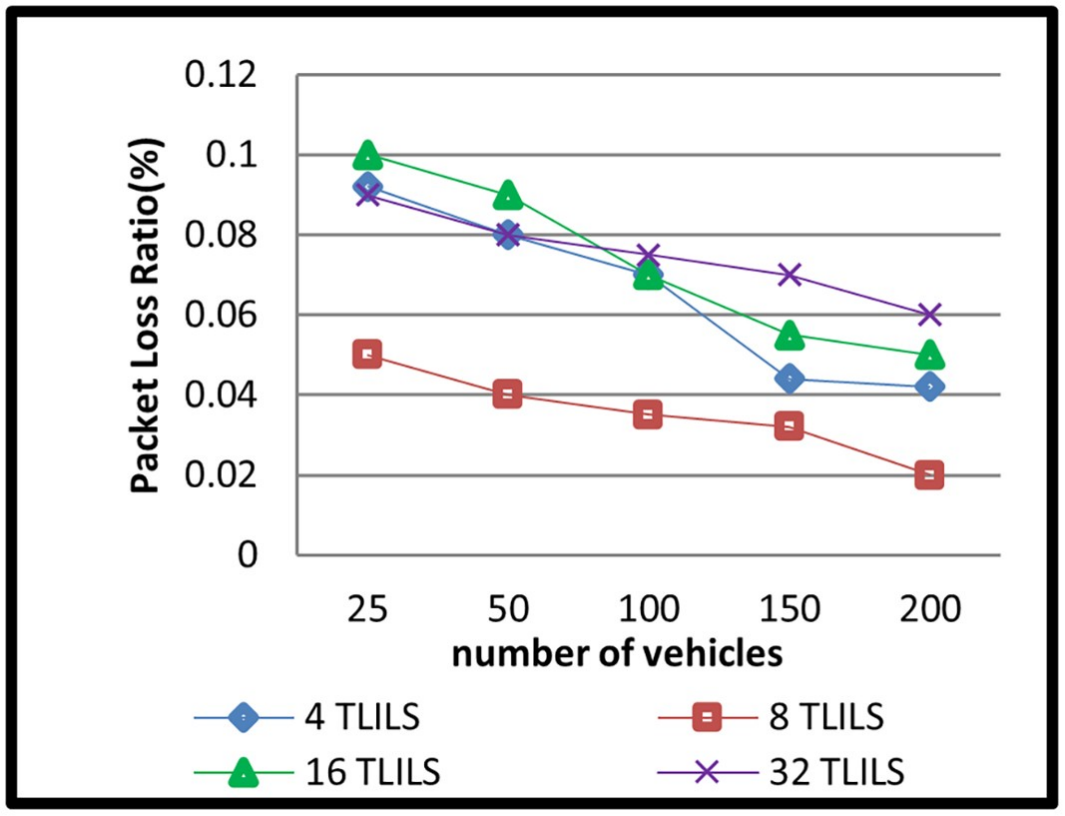
Packets loss ratio of information dissemination with the TLILS with 10 m/s speed.

**Fig 10:**
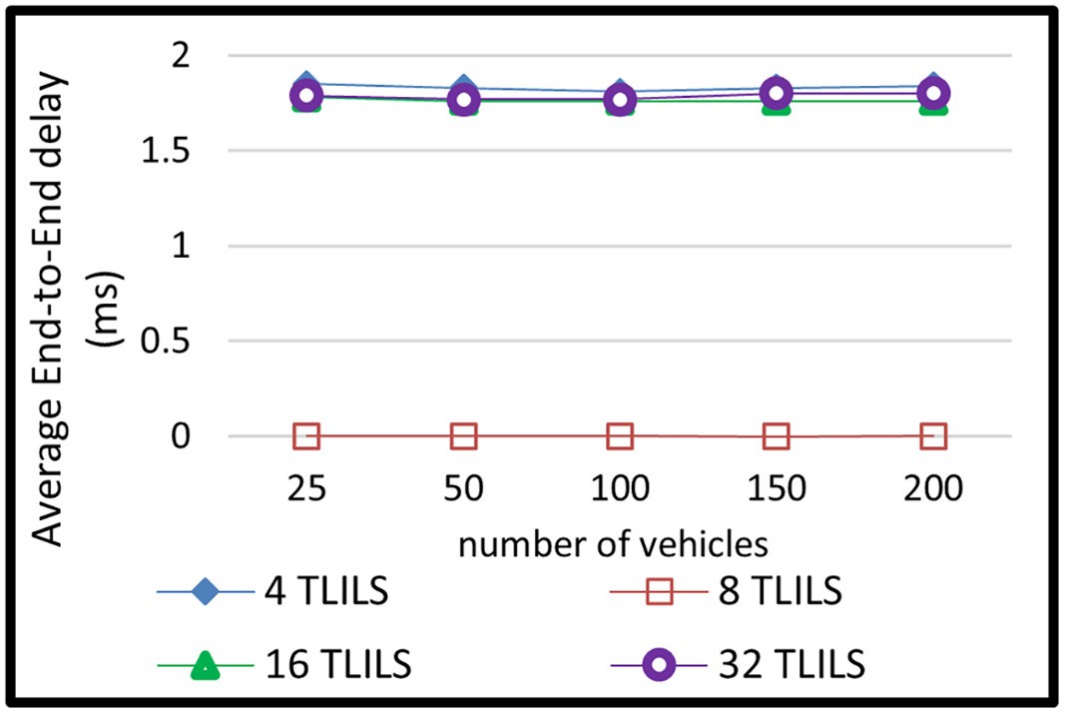
End-to-end delay of information dissemination with the TLILS with 10 m/s speed.

**Fig 11:**
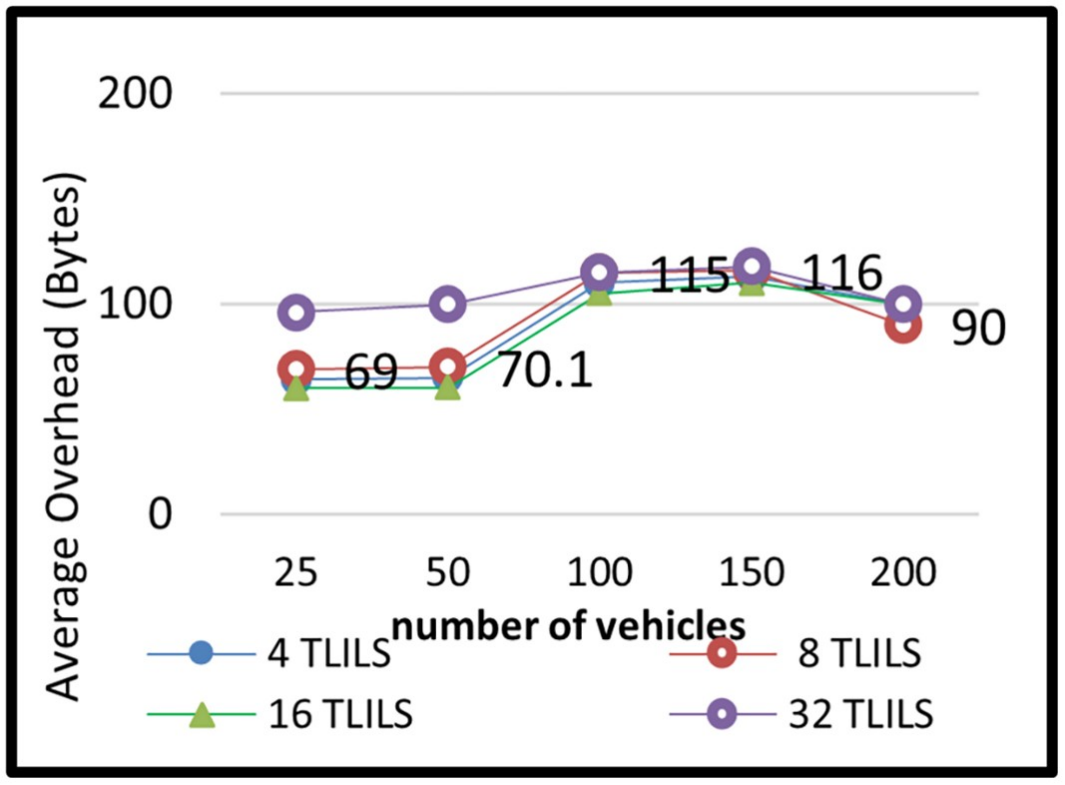
Overhead of information dissemination with the TLILS with 10 m/s speed.

The message dissemination in TLILS employing 4, 8, 16, and 32 TLILSs was tested in scenario 2 by altering the number of cars. The number of related cars in 5G VANETs has changed over time. In this simulation scenario, there were 20, 50,100,150, and 200 vehicles. The outcomes of this situation are depicted in Figs 8–11.

In the simulation, the beacon interval was one beacon per second. The average results of the ten simulation experiments are represented by each point. 25 vehicles represent the least number of vehicles. The low density was represented by 100 vehicles. A total of 150 vehicles were used to symbolize a high density. A total of 200 vehicles had the highest density.

The PDR is shown in Fig 8 by adjusting the number of TLILSs in a varied number of cars at a speed of 10 m/s. Using TLILS resulted in the largest PDR across all vehicles counts. When utilizing eight TLILSs, the PDR was 0.98. This is because eight TLILS is the optimized location server choice for many parameters such as speed. However, when using 4, 16, or 32 TLILSs, the PDR is lower. When using four TLILSs, localization fails in response to the vehicle of interest when it is beyond the wireless broadcast range.

According to the simulation data, TLILS with eight TLILSs has the shortest PLR, which is less than 0.1 for all vehicle counts as shown in Fig 9. This is due to the use of numerous TLILSs for the position management service, which helps distribute the burden of position management data. TLILS with 4, 16, and 32 had a low PLR (less than 0.1). eight TLILS ensures the maximum traffic weight metric in the proposed location service.

By increasing the vehicle density to 200 vehicles, PLR decreased slightly in the TL-inspired location service. The more vehicles the more 5G VANET connections, but also the more overhead and packet collisions. The end-to-end delay time is caused by routing delays, including routing discovery, routing transmission, re-transmission, interface delay, and MAC delay. For all numbers of vehicles, using eight TLILSs supports the smallest delay (smaller than two milliseconds) as shown in Fig 10. This is since eight TLILS is the best location server option in terms of time.

The longer the delay time, the more the possibility of the packet is not transmitted. This is because of quick location server changes as in Fig 10. The location service is scalable because TLILS provides the smallest delay in a large density of vehicles. The small delay can be explained by the load distribution on multiple TLILSs at the intersections. TLILS also reduces the end-to-end delay by decreasing PLR. Because they do not support all road directions, using four TLILSs increases packet overhead. RLR increases the end-to-end delay in ZGLS. eight TLILS is the best location server option in terms of time. eight TLILS ensures the maximum traffic weight metric in the proposed location service.

Fig 11 depicts an overhead in multiple numbers of vehicles at a speed of 10 m/s. Results show that TLILS using 16 and 32 TLILSs in intersections gives the lowest overhead over all other numbers of TLILSs in all densities in intersections (smaller than 100 bytes). At intersections, load distribution across many TLILSs saves overhead.

The overhead decreases at a high number of vehicles as a search query for location and messages are sent in the same transmission. TLILS uses roadside units to help to forward in message dissemination and positions directly to the destination vehicle which reduces overhead. eight TLILS is the best location server option in terms of density. eight TLILS ensures the maximum traffic weight metric in the proposed location service. In TLILS, using four TLILSs is lower than the number of road directions. Using 8, or 16 TLILSs is high increases overhead, particularly in high speeds of vehicles because of the large errors in routes.

## 6. Conclusion

Localization service in 5G VANETs faces several challenges. Localization service must be stable, scalable, varying at application types, helping forward information dissemination, and positions. Proposed TLILS used in this paper to use four RSUs at a junction in all directions to locate destination vehicles TLILS stores beacons from its neighbors to provide a location administration service. TLILS surpasses BBR and ZGLS for PDR and end-to-end latency, according to the findings. The findings of the three situations demonstrate that one TLILSs in one intersection with relays in slow traffic is making a precise determination of the location. It also shows that the ideal number of TLILSs is half of the total number of intersections on the map. In the future, a traffic light can be exploited more in VANETs. Traffic light may help in RSUs, and vehicles jobs.
